# Lipoprotein-associated phospholipase A2 and complications of diabetes mellitus: a systematic review and meta-analysis

**DOI:** 10.3389/fendo.2026.1765261

**Published:** 2026-02-12

**Authors:** Sujuan Guo, Xiaojuan Zhao

**Affiliations:** Department of Endocrinology Medicine, The First People’s Hospital of Lianyungang, Lianyungang, Jiangsu, China

**Keywords:** cardiovascular disease, complications, diabetic nephropathy, hyperglycemia, inflammation

## Abstract

**Objective:**

This systematic review aimed to assess whether lipoprotein-associated phospholipase A2 (Lp-PLA2) is associated with increased risk of complications in patients with diabetes mellitus (DM).

**Methods:**

We searched PubMed, Embase, Web of Science, and Scopus databases for studies fulfilling the inclusion criteria till 30 June 2025. The relationship between Lp-PLA2 and cardiovascular disorders (CVD), diabetic kidney disease (DKD), diabetic retinopathy (DR), diabetic neuropathy (DN), and lower extremity arterial disease (LEAD) was assessed using a qualitative and quantitative analysis.

**Results:**

Twelve studies were included. The majority of studies were on DKD. Pooled analysis showed that high Lp-PLA2 was associated with significantly higher risk of DKD (OR: 1.01 95% CI: 1.01, 1.02 I^2^ = 93%) but not for CVD (OR: 1.11 95% CI: 0.97, 1.26 I^2^ = 88%), DN (OR: 2.02 95% CI: 0.40, 10.23 I^2^ = 88%) or DR (OR: 1.28 95% CI: 0.49, 3.34 I^2^ = 96%). Sensitivity analysis revealed non-significant results for DKD and CVD. Subgroup analysis for DKD showed that heterogeneity reduced to zero in cross-sectional studies, among those with <30% prevalence of DKD, and among those reporting adjusted data, but results also became non-significant across multiple subgroups.

**Conclusions:**

Limited evidence indicates that high Lp-PLA2 may be predictive of DKD in DM patients. However, the strength of the association is too low and the finding may not be relevant for clinical application. Lp-PLA2 was not found to predict CVD, DN, or DR, but with very scarce data. The high heterogeneity and non-significant results on sensitivity analysis limits the strength of the evidence. More robust studies are required to supplement the present evidence.

**Systematic Review Registration:**

https://www.crd.york.ac.uk/prospero/, PROSPERO CRD420251069254.

## Introduction

Diabetes Mellitus (DM) is a common chronic metabolic condition that causes organ damage as a direct or indirect consequence of chronic hyperglycemia. Its prevalence in those aged 20 to 79 years was estimated to be 10.5% in 2021, and it is expected to climb to 12.2% by 2045, owing to an aging population, sedentary lifestyle, and poor diet (1). The condition also results in considerable healthcare costs, with 966 billion USD in 2021 and a projected increase to 1,054 billion USD by 2045 (2). Despite the complexities of DM’s pathogenesis, various risk factors have been found that potentially enhance the possibility of disease development (3). Adjustable risk factors include nutrition, physical activity, and obesity, while non-adjustable risk factors include age, ethnicity, concomitant diseases, familial and genetic susceptibility (4). Diverse clinical presentations and disease progressions are common among patients with DM, which can lead to diagnostic delays, a variety of pathophysiological abnormalities, and varying susceptibilities to complications (5). Macrovascular and microvascular complications, namely cardiovascular disorders (CVD), diabetic kidney disease (DKD), diabetic retinopathy (DR), lower extremity arterial disease (LEAD), and diabetic neuropathy (DN) can cause significant disability, impaired quality of life, and increased mortality among DM patients. Throughout their illness, most DM patients will display one or more of these as overt or subclinical symptoms, which results in a significant patient burden (6). Given the crippling implications, preventive strategies such as modifying cardiovascular risk factors, rigorous glucose management, patient education, and low-cost screening have been proposed (7). Furthermore, various blood biomarkers have also been studied for predicting the likelihood of DM complications, to allow early detection and risk stratification (8). Nevertheless, no robust blood-based marker has been globally accepted to date.

Lipoprotein-associated phospholipase A2 (Lp-PLA2) is a circulating enzyme with antioxidant and pro-inflammatory properties. It is a new marker linked to low-density lipoprotein cholesterol, atherosclerosis, and coronary heart disease (9, 10). Lp-PLA2 is largely released by macrophages, monocytes, and other inflammatory cells and attaches to LDL cholesterol. It also binds to HDL cholesterol and lipoprotein(a) to a lesser extent. LDL cholesterol acts as a reservoir for Lp-PLA2, which is not activated until it is oxidized (11). Lp-PLA2 hydrolyzes phospholipids during inflammation and oxidative stress, producing proinflammatory lipid mediators such as lysophospholipids and oxidized free fatty acids, which promote atherosclerosis. Lp-PLA2, thus, plays a role in the progression of inflammation, namely in vascular inflammation and atherosclerosis (12, 13). Although Lp-PLA2 is a well-known marker for large vessel disease (9), its relationship with microvascular illnesses is not fully understood. Complications of DM are primarily microvascular or macrovascular in origin, and therefore, Lp-PLA2 can be a novel and potential marker for screening patients. While a number of studies have reported associations between Lp-PLA2 and DM complications in the past, evidence remains unclear and incoherent. We therefore conducted this first systematic review and meta-analysis of the literature to examine the role of Lp-PLA2 in predicting DM complications.

## Materials and methods

This meta-analysis followed PRISMA guidelines (14) and was registered with PROSPERO (CRD420251069254). The four databases that were examined from their inception until June 30, 2025, were PubMed, Embase, Web of Science, and Scopus. In **Supplementary Table 1**, the comprehensive search approach for each database to identify pertinent studies for the review is documented. Two reviewers completed the search separately, with no language or date constraints.

The searched studies were to be cross-sectional, cohort, case-control in design, or secondary analysis of a randomized controlled trial. Studies should have focused on a “population” of DM patients. The “exposure” was high levels of serum Lp-PLA2. The “Comparative” group included those with low levels of serum Lp-PLA2. As “Outcomes”, qualified studies were to provide an adjusted relationship between high Lp-PLA2 and DM complications. Based on preliminary searches, we identified DKD, DN, DR, CVD, and LEAD as DM complications in our investigation.

We excluded studies published only in abstract form. We also eliminated studies not reporting separate outcomes for DM and those comparing the risk of complications between DM and healthy individuals. Editorials and reviews were also not eligible.

All databases’ search results were imported utilizing EndNote version X9 (Thomson Reuters, New York, NY, USA), and duplicates were automatically removed. The remaining records were checked individually by the two authors, and any differences were resolved through discussion. The screening process was carried out in two stages to determine the study’s relevance for this meta-analysis: (I) screening of the title and abstract, and (ii) screening of the full-text in accordance with the inclusion criteria to ascertain the study’s final suitability for both qualitative and quantitative analysis. Finally, we looked through the reference list of included research and Google Scholar for any missing studies.

Data was extracted into a standard Microsoft Word document by two review authors who operated independently. The retrieved data comprised the following: author, year, location, design, sample size, age, gender, diabetes duration, HbA1c, type of complication and its description, prevalence of the complication in the sample, Lp-PLA2 data management, follow-up, and adjusted covariates in the data analysis. Data was cross-checked, and differences between authors were resolved through discussions.

Two reviewers assessed the quality of the observational studies using the Newcastle Ottawa scale (15). The scale assesses bias in participant selection, comparison of exposure and control groups, and outcome assessment. Ratings can be anywhere from zero to nine, with higher ratings meaning better quality. Detailed discussions were conducted to resolve any discrepancies.

After acquiring the outcome data from the research, it was categorized according to the type of complication. The DerSimonian and Laird random-effects meta-analysis model was then used to obtain the pooled odds ratios (OR) with 95% confidence intervals (CI) for each complication. Data was analyzed using “Review Manager” software (version 5.3). The heterogeneity of studies was analyzed using Cochran’s Q statistic and the I^2^ index. Significant heterogeneity was indicated by an I^2^ of more than 50% or a P-value < 0.05. Due to the minimal number of studies available, we did not create funnel plots. Sensitivity analysis was performed by removing one study at a time from the meta-analysis for outcomes with more than three studies. Subgroup analysis was conducted only for DKD based on study design, sample size, prevalence of DKD, and type of data.

## Results

**Figure 1:**
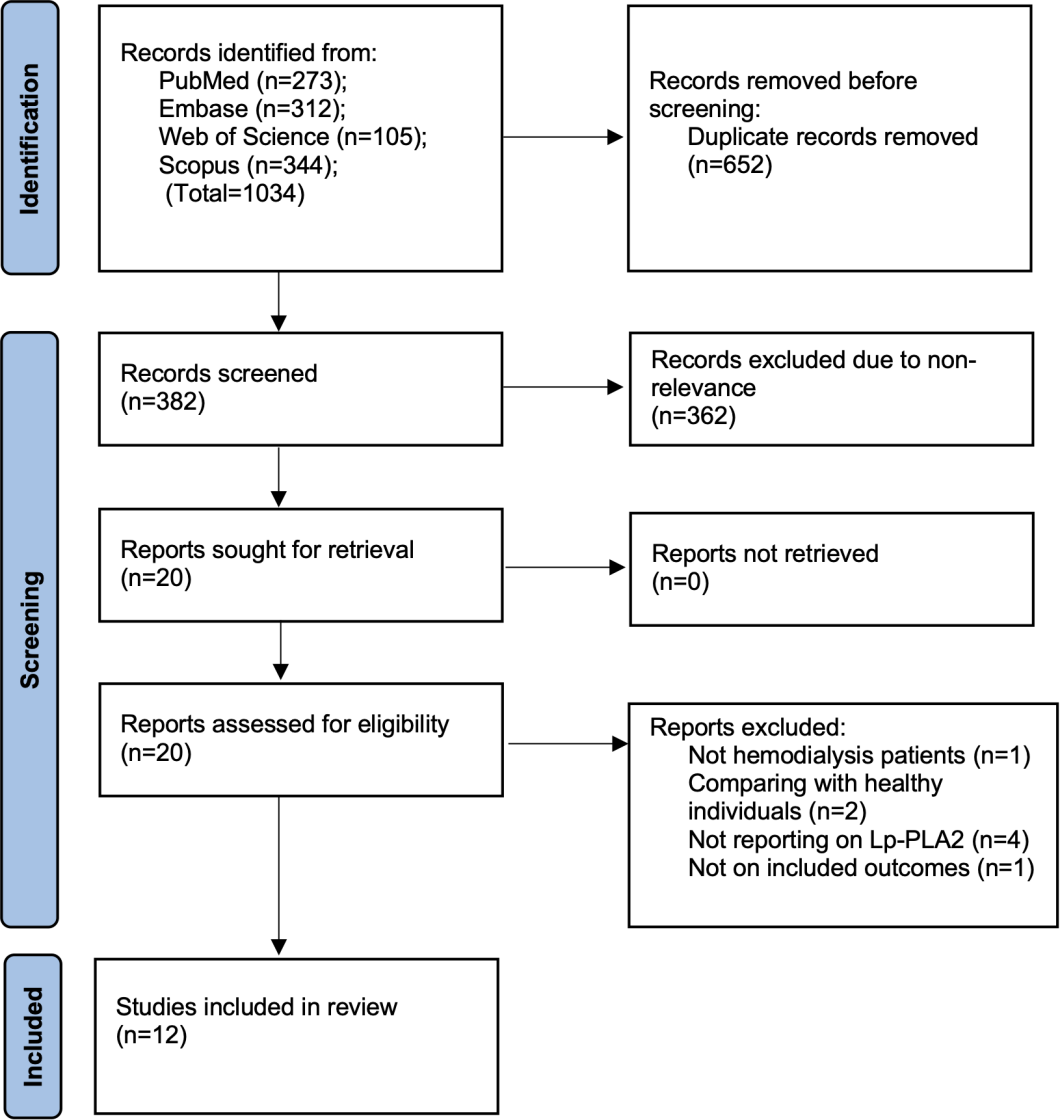
Study flowchart.

**Figure 1** depicts the findings of the literature search as well as the studies that were rejected at each stage of screening. The four databases contained one thousand thirty-four studies. However, just 382 of these were unique. The reviewers’ meticulous screening resulted in the exclusion of the majority of papers because they were not relevant to the current analysis. Twenty studies underwent full-text screening, and 12 were included (16–27). There was no disagreement over the inclusion of any study.

Data extracted from the studies is shown in **Table 1**. The included studies were published over the past 15 years. The country of origin was mostly China, which contributed to nine of the 13 studies. The remaining were from the USA, UK, and Germany. Three studies were cross-sectional in design, while ten were retrospective cohort studies. The minimal sample size was 96 while the maximum was 1517, resulting in a pooled sample size of 8731 patients. In all studies, males predominated in terms of gender. DM duration and baseline HbA1c varied between studies. Except for two studies, all studies reported adjusted associations between Lp-PLA2 and DM complications. However, the variables used for adjustment varied between studies. Six studies reported data on DKD, of which one study also reported unadjusted data for DN and DR. In addition, one study each was available for DN and DR. Three studies reported associations between Lp-PLA2 and CVD. There was only one study available for LEAD. Definitions of complications reported by the studies are mentioned in detail in **Table 1**. The majority of studies used Lp-PLA2 as a continuous variable, with a few studies dividing the data into quartiles or tertiles.

**Table 1:** Details of included studies.

Study	Country	Design	Sample size	Mean age (years)	Male	DM duration (years)	HbA1c (%)	Complication	Definition	% with complication	Lp-PLA2 data	Follow-up (years)	Adjusted covariates
He 2025 (20)	China	RC	880	61.1±12.2	61.8	10.4± 8.1	8.9± 2.1	DN	Based on neurological examination	58.6	Quartiles	NR	Age, sex, BMI, diabetes duration, hypertension, stroke, heart disease, FPG, fasting C-peptide, 2-h C-peptide, TC, TG, LDL, eGFR and Scr
Zhang 2024 (22)	China	RC	422	52.7± 13.5	61.6	6.7± 8.2	9± 2.4	DKD	UAER≥ 300mg/24h	37.7	Continuous	NR	Age, smoking, BMI, blood pressure, WBC count, SII, eGFR, Scr, uric acid, retinol binding protein, C-peptide, FBG, and TG-glucose
Xing 2024 (24)	China	RC	80	56.2± 11.5	58.7	NR	NR	DKD	UACR≥30 mg/g	40	Continuous	NR	Age, FBG, TG, TC
Chen 2024 (27)	China	RC	979	57(52-61)	65.9	NR	6.5 (5.5–7.4)	CVD	Cardiovascular death, acute coronary syndrome, coronary stent implantation	11.8	Continuous	84 months	Age, sex, low- density lipoprotein cholesterol, high-density lipoprotein cholesterol, apolipoprotein A-I
Zhai 2023 (25)	China	RC	342	63.9± 11	55	7.6± 4.1	7.5± 1.1	DKD	UACR≥30 mg/g	33.3	Continuous	NR	NR
Yang 2021 (18)	China	CS	915	50± 11	55.7	NR	NR	DKD	eGFR≦̸ 60ml/min/1.73 m^2^	17.9	Continuous	Up to 5 years	NR
Xu 2020 (16)	China	RC	519	63.9± 13.6	57.8	9.9± 8	8.9± 2.2	LEAD	Ankle-brachial index < 0.91	33.3	Continuous	NR	Age, sex, BMI, diabetes duration, smoking hypertension, HBA1c, TC, TG, LDL cholesterol, high-density lipoprotein cholesterol, eGFR
Seyfarth 2019 (19)	Germany	CS	165	17± 2.3	60.6	14.2± 2	8.2± 1.3	DKD	Albuminuria*	13.9	Continuous	NR	Sex, age, BMI, HbA1c, insulin dosage, blood pressure, TC and high-density lipoprotein cholesterol
Hu 2019 (17)	China	CS	1452	58.5± 12.5	61.6	7.1± 6.8	9.1± 2.1	DKD and unadjusted data for DN, DR	eGFR≦̸ 60ml/min/1.73 m^2^ or UAER≥30 mg/24h	40.3	Continuous and categorical	NR	Age, gender, duration of diabetes. HbA1c, BMI, TC, high density lipoprotein cholesterol, TG, LDL cholesterol, presence of CAD and carotid plaque, use of statins, and blood pressure
Siddiqui 2018 (26)	UK	RC	1364	67± 11	59	9± 7	7.6± 1.6	DR	Clinical records	59.8	Quartiles	3 years	Age, sex, diabetes-controlling medication, use of statins, HbA1c levels, systolic blood pressure, LDL
Miller 2010 (21)	USA	RC	96	30± 5.6	50	22 ± 5.6	8.9± 1.5	CVD	CAD death, MI and/or Q-waves, ischemic ECG, physician diagnosed angina	42.7	Continuous	Up to 18 years	CRP, diabetes duration, sex, LDL-cholesterol, systolic blood pressure, blood pressure medication use, white blood cells
Hatoum 2010 (23)	USA	RC	1517	60± NR	51.2	8± NR	7 ± NR	CVD	CABG or percutaneous transluminal coronary angioplasty, nonfatal myocardial infarction, and fatal heart disease	15.4	Tertiles	10 years	Age, fasting status, smoking, alcohol intake, physical activity, duration of diabetes, aspirin use, cholesterol-lowering medication use, family history of myocardial infarction, history of hypertension, BMI, HDL, HbA1c, CRP, intercellular adhesion molecule, insulin use, waist circumference, eGFR, and hormone replacement therapy use (women only)

TC, total cholesterol; TG, triglyceride; eGFR, estimated glomerular filtration rate; Scr, serum creatinine; FPG, fasting blood glucose; BMI, body mass index; DN, diabetic neuropathy; DKD, Diabetic kidney disease; UAER, urinary albumin excretion rate; UACR, urinary albumin/creatinine ratio; BMI, body mass index; SII, systemic immune-inflammatory index; NR, not reported; RC, retrospective cohort; CVD, cardiovascular disease; CS, cross-sectional; LEAD, lower extremity arterial disease; LDL, low density lipoprotein; CRP, C-reactive protein; CAD, coronary artery disease; CABG, coronary artery bypass graft surgery.

*defined as urine albumin concentration between 20 and 200 mg/l, as urine albumin-to-creatinine ratio between 2.5 and 25 mg/mmol creatinine (male) or between 3.5 and 25 mg/mmol creatinine (female), or as a urine albumin excretion rate of 20–200 lg/min in a timed urine collection or 30–300 mg/24 hours in a 24-hour urine collection.

Quality assessment of studies based on the two authors’ judgment is shown in **Table 2**. Variability was noted in awarding of points for comparability and outcome assessment, especially for follow-up time. Studies not reporting adjusted data were not given points for comparability. The total NOS score of two studies was 6, seven studies were 8, and four studies were nine.

**Table 2:** Newcastle Ottawa scale scores of studies.

Study	Selection	Comparability	Outcome assessment	Total
He 2025 (20)	4	2	2	8
Zhang 2025	4	2	2	8
Xing 2024 (24)	4	2	2	8
Chen 2024 (27)	4	2	3	9
Zhai 2023 (25)	4	–	2	6
Yang 2021 (18)	4	–	2	6
Xu 2020 (16)	4	2	2	8
Seyfarth 2019 (19)	4	2	2	8
Hu 2019 (17)	4	2	2	8
Siddiqui 2018 (26)	4	2	3	9
Miller 2010 (21)	4	2	3	9
Hatoum 2010 (23)	4	2	3	9

### DKD

**Figure 2:**
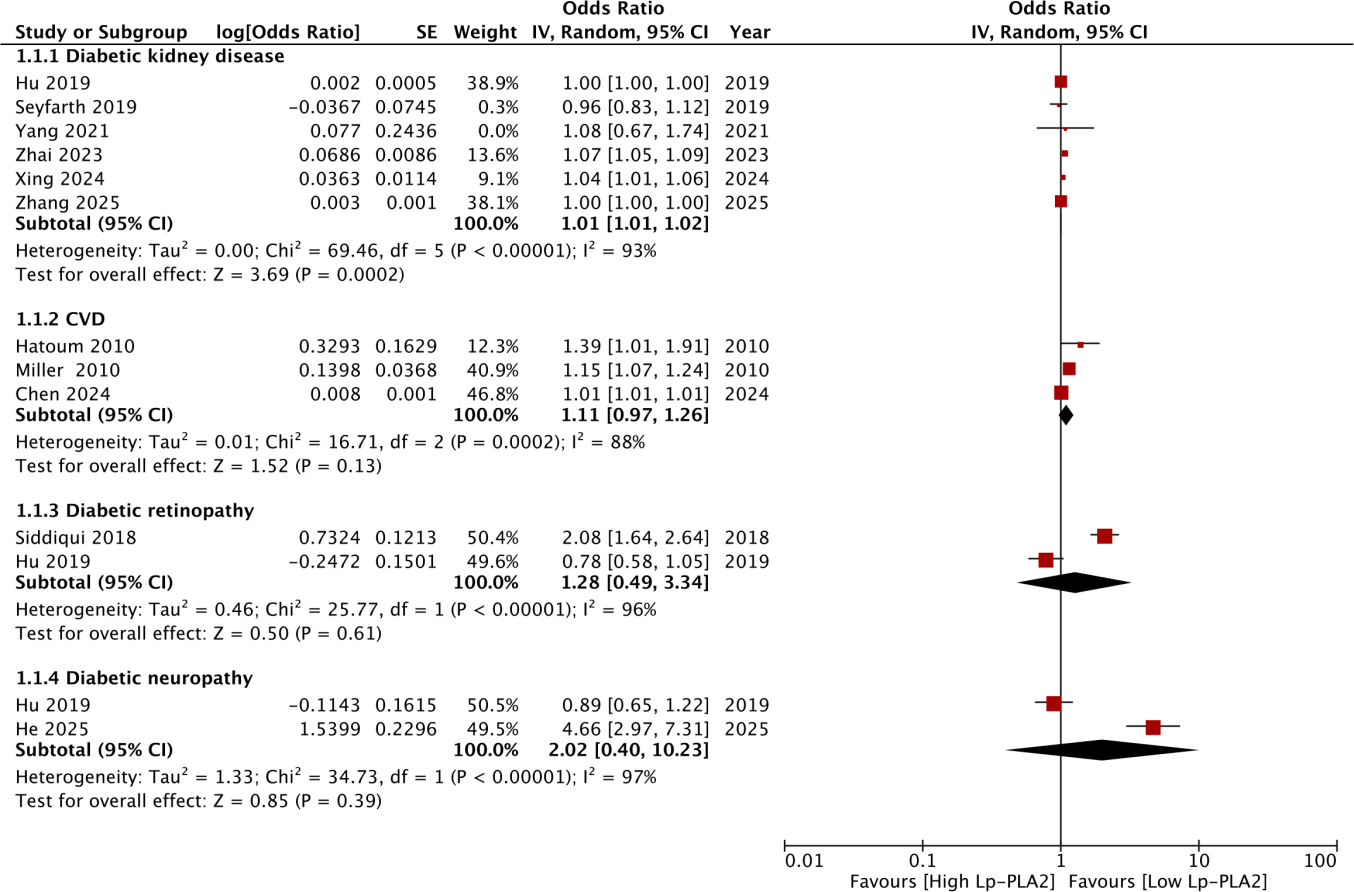
Meta-analysis of the association between high Lp-PLA2 levels and diabetic complications with subgroup analysis for DKD, CVD, DR, and DN.

**Figure 3:**
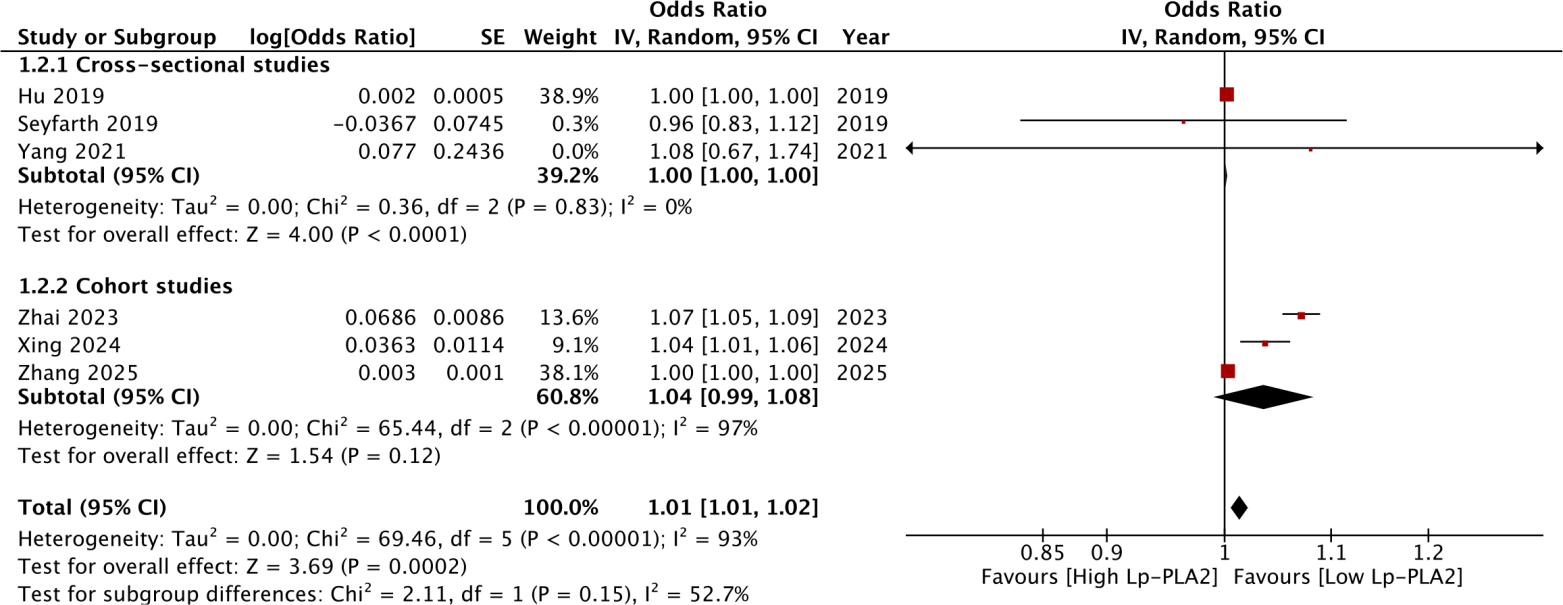
Subgroup analysis of the association between high Lp-PLA2 levels and DKD based on study design.

**Figure 2** shows the meta-analysis of all DM complications analyzed in the present review. Pooled analysis showed that high Lp-PLA2 was associated with significantly higher risk of DKD (OR: 1.01 95% CI: 1.01, 1.02 I^2^ = 93%). Sensitivity analysis showed that the results turned non-significant on the exclusion of two studies and heterogeneity remained persistently high (**Table 3**). Subgroup analysis results are shown in **Table 4**. Results were significant for cross-sectional studies but not for cohort studies (**Figure 3**). Segregation based on sample size also led to non-significant results. However, results remained significant depending on the prevalence of DKD and the type of data (crude or adjusted). Heterogeneity reduced to zero for cross-sectional studies, for those with <30% prevalence of DKD, and those reporting adjusted data.

**Table 3:** Sensitivity analysis results.

Excluded study	Re-calculated odds ratio [95% CI]	I^2^
DKD		
Seyfarth 2019 (19)	1.01 [1.01, 1.02]	94
Hu 2019 (17)	1.03 [0.99, 1.08]	94
Yang 2021 (18)	1.01 [1.01, 1.02]	94
Zhai 2023 (25)	1.01 [1.01, 1.02]	60
Xing 2024 (24)	1.01 [1.00, 1.02]	93
Zhang 2025	1.03 [0.99, 1.08]	94
CVD		
Hatoum 2010 (23)	1.07 [0.94, 1.22]	92
Miller 2010 (21)	1.14 [0.84, 1.54]	74
Chen 2024 (27)	1.18 [1.04, 1.35]	22

DKD, diabetic kidney disease; CVD, cardiovascular disease; CI, confidence intervals.

**Table 4:** Subgroup analysis for DKD.

Variable	Groups	Studies	Odds ratio [95% CI]	I^2^
Study design	Cross-sectional Cohort	3 3	1.00 [1.00, 1.00] 1.04 [0.99, 1.08]	0 97
Sample size	>200 <200	3 3	1.04 [0.97, 1.10] 1.02 [0.99, 1.05]	97 77
Prevalence of DKD	>30% <30%	3 3	1.01 [1.00, 1.02] 1.04 [1.01, 1.06]	97 0
Type of data	Adjusted data Crude data	4 2	1.00 [1.00, 1.00] 1.05 [1.02, 1.09]	0 80

CI, confidence intervals; DKD, Diabetic kidney disease.

### CVD

Meta-analysis of three studies showed that high Lp-PLA2 was not significantly associated with CVD in DM patients (OR: 1.11 95% CI: 0.97, 1.26 I^2^ = 88%). Sensitivity analysis showed that results turned significant on the exclusion of Chen et al (27). On descriptive analysis, it was noted that the study of Chen et al (27) in their retrospective study of 979 DM patients recorded 116 CVD events during a follow-up period of 84 months. They noted that Lp-PLA2 was a significant predictor of CVD events but with a small effect size (OR: 1.008 95% CI: 1.006, 1.010). Miller et al (21) in their small retrospective study of 96 patients found that Lp-PLA2 was a significant predictor of CVD events on univariate analysis (OR: 1.53 95% CI: 1.11, 2.12), however, the significance was lost on adjustment of multiple confounders (OR: 1.15 95% CI: 0.70, 1.87). On the other hand, Hatoum et al (23) in one of the earliest studies on 740 men and 777 women found that even after adjustment of multiple confounders, Lp-PLA2 was a significant predictor of CVD events (OR: 1.39 95% CI: 1.01, 1.90) in both men and women.

### Other complications

Only two studies reported data on DN and DR and pooled analysis failed to show a statistically significant association between high Lp-PLA2 and DN (OR: 2.02 95% CI: 0.40, 10.23 I^2^ = 88%) or DR (OR: 1.28 95% CI: 0.49, 3.34 I^2^ = 96%). Of the two studies included in both analyses, one study of Hu et al. reported only unadjusted data and noted no association between Lp-PLA2 and DN or DR. On the other hand, He et al (20) found that Lp-PLA2 was a significant and strong predictor of DN in their retrospective analysis of 880 patients (OR: 4.66, 95% CI: 2.97, 7.31). Likewise, the study of Siddiqui et al (26) in their retrospective analysis of 1364 patients with follow-up of up to three years found that Lp-PLA2 was a significant and strong predictor of DR (OR: 2.08, 95% CI: 1.64, 2.64). However, in both these studies, Lp-PLA2 was divided into quartiles and the results were compared between the highest quartile with the lowest.

Lastly, only one study examined the association between Lp-PLA2 and LEAD. Xu et al (16) in a retrospective analysis of 519 patients showed that Lp-PLA2 levels in the LEAD group were significantly higher than non-LEAD group. Adjusted data analysis also revealed that Lp-PLA2 was a statistically significant predictor of LEAD in DM patients (OR: 1.018 95% CI: 1.007–1.029).

## Discussion

The present investigation is the first review in the literature to examine the association between Lp-PLA2 and DM complications. The majority of available studies were on DKD while a limited number of studies reported data on CVD, DN, DR and only one study was available on LEAD. A detailed meta-analysis and subgroup analysis were possible only on DKD, while for the remaining outcomes, a qualitative and quantitative analysis was conducted.

The pooled analysis showed that while high Lp-PLA2 was associated with increased risk of DKD, the overall effect size was very small. All included studies used Lp-PLA2 as a continuous variable and per-unit increase in Lp-PLA2 was associated with only 1% increase in the risk of DKD. Therefore, despite the statistical significant results, the clinical relevance of this finding may be very limited. With such minor association, Lp-PLA2 may not be suitable in routine clinical practice to predict DKD. Another limitation of the analysis was the instability of the results in the sensitivity analysis, as the association turned non-significant on the exclusion of two studies. The high inter-study heterogeneity in the meta-analysis is a cause of concern, and we recommend extreme caution in the interpretation of the results which do not permit strong conclusions. The possible reasons for such high inter-study heterogeneity can be several and could be due to differences in patient ethnicity, study design, sample size, baseline DM control, DM duration, comorbidities, DKD definition, and follow-up. We conducted a detailed subgroup analysis depending on data availability, only to find that while inter-study heterogeneity decreased to zero for several subgroups, summary estimates for several of these subgroups became non-significant. Of note, no inter-study heterogeneity was noted in cross-sectional studies, those with a lower prevalence of DKD, and for studies reporting adjusted estimates. However, heterogeneity remained high for other subgroups. Other notable findings in the subgroup analysis were that the results were non-significant for cohort studies but remained significant for cross-sectional studies. The evidence generated by cohort studies is better than that of cross-sectional studies as they enable researchers to determine temporality and evaluate disease incidence over time, which is essential for comprehending cause-and-effect relationships. Cross-sectional analysis, on the other hand, can identify associations between variables, but it cannot determine cause-and-effect relationships (28). In particular, reverse causation cannot be ruled out, especially in DKD, where declining renal function could itself affect Lp-PLA2 levels via changes in inflammation, oxidative stress, or clearance (18). Cross-sectional studies cannot establish whether high Lp-PLA2 levels precede complications or are a consequence of established disease, so their results should be interpreted carefully.

A second important result of the subgroup analysis was that the effect size was marginally significant on the pooled analysis of adjusted data, but higher risk was noted when only crude estimates were combined. Development of DKD in DM patients has been associated with several key risk factors like age, uncontrolled blood sugar, hypertension, obesity, smoking, dyslipidemia, and physical inactivity (29, 30). While none of the included studies conducted a comprehensive adjusted of all possible risk factors due to paucity of baseline data, several of the included studies did include important covariates like baseline HbA1c, age, body mass index, hypertension, and dyslipidemia. Therefore, the summary estimates of adjusted data provide a better overview of the association between high Lp-PLA2 and DKD indicating that per-unit increase may not be highly predictive of the complication.

The pooled analysis also showed that high Lp-PLA2 was not associated with higher risk of CVD, DN or DR. A single study also demonstrated an increased risk of LEAD with high Lp-PLA2 levels but herein too the effect size was very small with only a 1.8% increase in the overall risk. On descriptive analysis, it was noted that the study of Hatoum et al (23), Siddiqui et al (26), and Hu et al (17) found a significant and large association between high Lp-PLA2 and CVD, DR and DN respectively. On the contrary, other studies reporting data on these complications found a minimal increase in risk or even no significant associations between Lp-PLA2 and DM complications. A possible reason for such discrepancy is that the studies of Hatoum et al (23), Siddiqui et al (26), and Hu et al (17) used Lp-PLA2 as a categorical variable while the remaining studies used it as a continuous variable. Analyzing Lp-PLA2 per unit assumes a linear, consistent risk increase across all levels, which might underestimate significant risks if the relationship is threshold-based or nonlinear. This probably explains the very small effect sizes in our pooled results, where a 1-unit increase in Lp-PLA2 was associated with only a 1–2% increase in risk. Conversely, studies that compared the highest to the lowest categories consistently found larger, statistically significant associations, indicating that elevated Lp-PLA2 levels may carry more risk than variations across the entire range. Nevertheless, the category cut-points varied widely across studies, and there is currently no accepted clinical threshold for Lp-PLA2 in diabetic populations. In clinical practice, establishing an optimal cut-off of Lp-PLA2 can help screen patients for a higher risk of DM complications. However, the present evidence is scarce and more evidence is needed to justify its routine application in clinical practice.

In light of the current evidence, it is pertinent to mention that Lp-PLA2 has been found to be a predictor for other cardiovascular disorders. Li et al (31) in a systematic review and meta-analysis of 12 studies have shown that Lp-PLA2 when used as a categorical variable (highest category vs lowest category), significantly increases the risk of coronary heart disease and ischemic stroke by 46% and 58% respectively. However, a per-unit increase was associated with only a 12% increased risk of coronary heart disease, with no significant association with ischemic stroke. Likewise, studies also show that Lp-PLA2 is increased in patients with chronic kidney disease as compared to healthy controls (32).

The association between Lp-PLA2 and DM complications has been potentially linked to oxidative stress and inflammation. Lp-PLA2 is a novel inflammatory marker that plays a significant role in both microvascular and macrovascular events (13). It is essentially a platelet-activating factor acetylhydrolase that is secreted by monocyte macrophages, T lymphocytes, and mast cells. This enzyme is responsible for the degradation of platelet-activating factors (9). Increased levels of Lp-PLA2 can exacerbate oxidative stress and facilitate the apoptosis of endothelial cells. In a pro-inflammatory environment, Lp-PLA2 releases prostaglandins and leukotrienes, which induce endothelial cell dysfunction and stimulate the production of cytokines and adhesion factors. The disruption of endothelial function is regarded as an early event in renal microangiopathy, which serves as the basis for the subsequent DKD (33). Moreover, an independent relationship exists between urinary albumin excretion and a variety of inflammatory mediators, such as cytokines, chemokines, and adhesion factors, suggesting that they are essential in the pathogenesis of DKD (34). Secondly, Lp-PLA2 is also linked to the metabolism of lipoproteins, especially LDL (11). The oxidation of LDL and other lipoproteins has been demonstrated to be a critical factor in the development of atherosclerosis (13). The initiation and progression of atherosclerosis are fundamentally influenced by inflammation. Additionally, cardiovascular events and kidney injury have been closely associated with inflammation and atherosclerosis (11, 17, 35). Research has shown that Lp-Pla2 is a powerful predictor of cardiovascular events. Lyso-phosphatidylcholine and oxidized free fatty acids are two harmful vasoactive and inflammatory chemicals produced by Lp-Pla2 that encourage vascular wall damage and lesions increasing the risk of cardiovascular events (26). However, despite the strong connections between Lp-PLA2 and inflammation and atherosclerosis, the limited increased risk of DM complications with high Lp-PLA2 is puzzling and further clinical and basic research is needed to elucidate the relationship between the two.

We need to mention the limitations of the current analysis. Firstly, the number of studies for each complication was a major limitation. The scarce data only allowed a detailed analysis for DKD but not for other complications. Secondly, as mentioned earlier, inter-study heterogeneity is a concern. Despite conducting multiple subgroup analyses, several of the subgroups had high inter-study heterogeneity indicating that factors responsible for the same were not clearly identified. In this context, the evidence generated by the review should be interpreted with extreme caution till further robust studies are available.

Another factor contributing to heterogeneity is the variation in Lp-PLA2 measurement methods used across studies. Some studies employed mass–based assays, while others used activity–based assays. For this review, these measures were combined as indicators of the same biological pathway, despite their differences. Variations in assay type, platforms, calibration standards, and units could have led to differences in effect size and significance across studies. However, due to the limited number of studies, it was not possible to conduct assay-specific subgroup analyses. Thirdly, a subgroup analysis could be conducted only for DVD and we could not include all possible variables due to a lack of data. Additionally, there were variations in the definition of DKD and CVD which may have influenced outcomes. Fourthly, study designs available for analysis were either retrospective cohort studies or cross-sectional studies, both of which are prone to bias. Lastly, not all studies reported adjusted outcomes and even among those reporting adjusted estimates, the confounders varied. Several known confounders were missed which may have affected outcomes. Lastly, most of the studies included were conducted in Chinese populations, with only a few from Western cohorts. Differences in ethnicity and region, such as genetic background, cardiometabolic risk, inflammatory responses, lipid metabolism, access to healthcare, and treatment approaches, may affect baseline Lp-PLA2 levels and their association with diabetic complications. As a result, the modest effects observed, especially for DKD, might not apply to other populations. Care should be taken when applying these findings beyond East Asian groups, and these results should not be assumed to be universal without further validation.

## Conclusions

Limited evidence indicates that high Lp-PLA2 may be predictive of DKD in DM patients. Given the small strength of the association, the applicability of Lp-PLA2 in clinical practice to predict KD may be limited and not of clinical relevance. Lp-PLA2 was not found to predict CVD, DN or DR but with very scarce data. The high heterogeneity in the meta-analysis limits the strength of the evidence. Further prospective cohort studies with long-term follow-up using Lp-PLA2 as a categorical variable and reporting adjusted estimates are needed to provide superior quality evidence.

## Data Availability

Publicly available datasets were analyzed in this study. All analyses conducted are from published studies publicly available on PubMed, Embase, Web of Science, and Scopus databases.
