# Supplementary material for: Lipoprotein-associated phospholipase A2 and complications of diabetes mellitus: a systematic review and meta-analysis
Source: Front Endocrinol (Lausanne). 2026 Feb 12;17:1765261. doi: 10.3389/fendo.2026.1765261 (PMC12935637; doi:10.3389/fendo.2026.1765261)
Supplement: Supplementary file 1 [file Table1.docx]

**Supplemental Table 1: Search strategy**

**Embase**

1. ' diabetes mellitus'/exp OR ‘diabetes mellitus'

2. ‘Complications’

3. #1 AND #2

4. ‘Diabetic neuropathy’ OR ‘Diabetic nephropathy’ OR ‘Diabetic kidney disease’ OR ‘Diabetic retinopathy’ OR ‘diabetic cardiovascular disease’ OR ‘diabetes heart disease’ OR ‘diabetes coronary artery disease’ OR ‘diabetic lower extermity artherosclerosis’ OR ‘diabetic peripheral artery disease’

5. ‘Lipoprotein‐associated phospholipase A2’

6. #3 OR #4

7. #5 AND #6

**PubMed**

(((diabetes mellitus) AND (complications)) OR (((((((((Diabetic neuropathy) OR (Diabetic nephropathy)) OR (Diabetic kidney disease)) OR (Diabetic retinopathy)) OR (diabetic cardiovascular disease)) OR (diabetes heart disease)) OR (diabetes coronary artery disease)) OR (diabetic lower extermity artherosclerosis)) OR (diabetic peripheral artery disease))) AND (Lipoprotein‐associated phospholipase A2)

**Scopus**

((TITLE-ABS-KEY-AUTH(diabetes mellitus) AND (complications)) OR ((((((((TITLE-ABS-KEY-AUTH (Diabetic neuropathy) OR (Diabetic nephropathy)) OR (Diabetic kidney disease)) OR (Diabetic retinopathy)) OR (diabetic cardiovascular disease)) OR (diabetes heart disease)) OR (diabetes coronary artery disease)) OR (diabetic lower extermity artherosclerosis)) OR (diabetic peripheral artery disease))) AND (TITLE-ABS-KEY-AUTH(Lipoprotein‐associated phospholipase A2)

**Web of Science**

(((diabetes mellitus) AND (complications)) OR (((((((((Diabetic neuropathy) OR (Diabetic nephropathy)) OR (Diabetic kidney disease)) OR (Diabetic retinopathy)) OR (diabetic cardiovascular disease)) OR (diabetes heart disease)) OR (diabetes coronary artery disease)) OR (diabetic lower extermity artherosclerosis)) OR (diabetic peripheral artery disease))) AND (Lipoprotein‐associated phospholipase A2)
